# A short-arc hardware delay estimation method for inter-satellite links to improve BDS-3 precise orbit determination

**DOI:** 10.1038/s41598-025-02800-7

**Published:** 2025-05-23

**Authors:** Zhouming Yang, Yunbin Yuan, Bingfeng Tan

**Affiliations:** 1 State Key Laboratory of Precision Geodesy, Innovation Academy for Precision Measurement Science and Technology, CAS, Wuhan, 430077 China; 2 College of Earth and Planetary Sciences, University of Chinese Academy of Sciences, Beijing, China

**Keywords:** BDS-3 navigation satellite system, Inter-satellite link, Precise orbit determination, Hardware delay, Segmented Estimation, Aerospace engineering, Astronomy and planetary science

## Abstract

The inter-satellite link (ISL) equipment of the third-generation BeiDou Navigation Satellite System (BDS-3) can enhance the accuracy of precise orbit determination (POD) for BDS-3 satellites. However, the hardware delays within ISL observations can impact the measurement accuracy of ISL observations. This study evaluates the characteristics of hardware delays in ISL observations for BDS-3 medium Earth orbit (MEO) satellites and investigates their effects on POD. Analysis shows that most links exhibit slow variations in hardware delays, with standard deviations (STD) of 6.4 cm and 3.6 cm over 45 days for the C19-C25 and C20-C21 links, respectively. Conversely, some links like C30-C23 and C30-C33 show more pronounced variations, with STD values reaching 17.7 cm and 14.1 cm within three days. Links involving satellites C23 and C30 display higher variability and significant jumps in hardware delays compared with other links. The larger variations in hardware delays of these links can be absorbed by other parameters, thereby affecting the accuracy of parameter estimation. To mitigate their impact on POD, a segmented estimation strategy for hardware delays is proposed. This strategy divides a single link into multiple segments based on a set interval length and estimates the hardware delay parameters for each segment separately, thereby absorbing the variations in hardware delays or unmodeled errors. The effectiveness of this strategy is demonstrated through ISL POD residual analysis, comparison with L-band orbit determination results, and validation using Satellite Laser Ranging (SLR) residuals. The root mean square (RMS) of ISL observation residuals decreased by approximately 44.3%, from 6.1 cm to 3.4 cm. Comparisons with L-band orbits confirmed an approximate 28.2% reduction in the 3D RMS of orbit discrepancies, from 26.5 cm to 19.0 cm. Additionally, the RMS of the overall SLR residuals showed a slight decrease. Overall, the improved segmented estimation strategy effectively reduces the influence of hardware delay variations on BDS-3 orbit determination, notably enhancing the orbit determination accuracy of BDS-3 satellites, especially for C23 and C30, which experience larger hardware delay fluctuations.

## Introduction

The BeiDou-3 Navigation Satellite System (BDS-3) began providing global services in 2020, offering high-precision navigation, positioning, and timing services to users worldwide^1^. According to the most recent data, the BDS-3 constellation now includes 28 medium Earth orbit (MEO) satellites, 5 inclined geosynchronous orbit (IGSO) satellites, and 4 geostationary orbit (GEO) satellites^2^. To ensure continuous global service in the absence of sufficient overseas tracking stations, the BDS-3 system has prioritized the development and implementation of inter-satellite link (ISL) technology. ISL technology is valued for its ability to provide accurate distance measurements between BDS-3 satellites, offering distinct advantages over ground-based observations as it is not affected by tropospheric and ionospheric delays, thus more accurately reflecting the physical characteristics of the clocks on board the BDS-3 satellites^3,4^. Additionally, ISL technology enriches the inter-satellite observation data, enabling full-arc, high-precision orbit determination for BDS-3 satellites even when relying on a limited number of ground stations. However, ISLs are susceptible to a variety of complex errors, such as signal transmission delays, limitations in equipment precision, and environmental factors, which significantly complicate the processing of inter-satellite observation data. Therefore, there is a critical need for research into high-precision data processing algorithms and models for ISL technology.

The five new-generation BDS satellites (BDS-3 S) are equipped with inter-satellite observation devices based on the Ka-band^4–6^, which operate using a Concurrent Spatial Time Division Duplexing (CSTDD) system. The ISLs of the BDS-3 S system enable mutual communication between satellites, enabling the constellation to achieve joint orbit determination and time synchronization^7^. To evaluate the orbit determination accuracy of these experimental satellites, Tang et al. (2018) conducted tests comparing the BDS-3 S satellite orbits through overlapping orbit analysis and satellite laser ranging (SLR) validation. The results indicated that inter-satellite ranging can effectively improve the orbital accuracy of BDS-3 S satellites, with the radial precision of MEO satellites reaching 10.0 cm. Following the BDS-3 S satellites, the BDS-3 satellites, designed for global service, were subsequently launched, all equipped with ISL equipment. To further assess the improvements in the orbit and clock offset determination of BDS-3 satellites achieved through inter-satellite ranging, Xie et al. (2020) analyzed the ISL data from BDS-3 MEO and GEO satellites. The analysis revealed that the radial orbit errors for MEO satellites, as determined by Ka-band measurements, ranged from 2 to 4 cm, while those for GEO satellites were 8 to 10 cm. Additionally, ISL technology enhances the accuracy of broadcast ephemerides^8^, resulting in more than 98% of ephemeris parameters and 93% of clock parameters being updated within one hour when combining ground stations and ISLs^9^. As the BDS-3 progressively reaches full operational capability, researchers have undertaken a wide range of studies focused on POD utilizing ISLs. These investigations encompass several key areas: the development and optimization of orbit determination algorithms for BDS-3 satellites^10^; the examination of overall constellation self-rotation^11–15^; the improvement of solar radiation pressure models specific to BDS-3 satellites^16,17^; the POD of GEO BDS-3 satellites^18^; the estimation of satellite clock offsets within the BDS-3 constellation; and the assessment of ISL observation quality^18–24^. Through these comprehensive research endeavors, significant insights into enhancing the accuracy and reliability of BDS-3 POD have been gained.

In existing ISL POD, the ISL hardware delay parameters were treated as satellite-specific, with one clock-free (CF) combination hardware delay parameter estimated for each satellite over a 72-hour period. However, the assumption that ISL hardware delays are satellite-related constant parameters implies that these delays do not vary with the specific ISL link. If satellite-related hardware delays differ between various ISL links, it suggests that the satellite-specific hardware delay parameters would vary when different ISL links are formed^25^. To address this issue, Wang et al. (2019) designed two strategies for estimating hardware delays: one involves estimating satellite-related hardware delays within the POD process; the other entails estimating the hardware delays for individual ISL links during POD. The effectiveness of these two strategies was evaluated using ISL residuals and operational orbit determinations (OODs). The evaluation results indicate that, excluding the satellites C29 and C30, the strategy of estimating hardware delays for individual ISL links performs better than estimating satellite-related hardware delays. This finding underscores the importance of accounting for variability in hardware delays across different ISL links to improve the accuracy of POD. Moreover, there are differences in the observational noise among various links, especially those involving satellites C23 and C26, which exhibit higher noise levels compared to other MEO satellites^19^. These findings suggest that estimating hardware delay parameters per satellite may not be the optimal approach due to variations in link measurement noise. Hence, it is essential to investigate the variations in CF combination hardware delays for each ISL of the BDS-3 satellites. Based on the research by Wang et al. (2019), this study applies a segmented estimation approach to the CF combined hardware delays of individual ISL links. Furthermore, it evaluates the effects of different estimation strategies on POD.

To study the hardware delays of the CF combinations of BDS-3 satellites, we integrate precise ephemerides with raw ISL observational data to estimate the CF combination hardware delay parameters for each link and analyze the characteristics of these parameters’ variations. Based on the characteristics of hardware delay changes, multiple segmented estimation strategies for the hardware delays of single links are designed. The effectiveness of the segmented hardware delay estimation strategies is evaluated using ISL orbit determination residuals, comparisons with L-band orbits, and SLR validation. The structure of this paper is organized as follows: First, we introduce the BDS-3 ISL observation model and the results of the analysis of CF combination hardware delays. Second, we present the status of ISL establishment and the segmented estimation strategies for ISL hardware delays, followed by an analysis and discussion of the orbit determination results. Finally, we summarize the findings and provide a conclusion.

## ISL observation model and hardware delay Estimation

This section first presents the observation model for the BDS-3 ISLs. It then evaluates the quality of the ISL signals. During the assessment of ISL signal quality, a comprehensive comparison of the differences among various links is conducted to elucidate the characteristics of these signals.

### Basic BDS-3 ISL observation model

The original observation model for the ISLs can be expressed as:

1 $$\begin{gathered} {P_{AB}}\left( {{t_1}} \right)=\left| {{{\overset{\lower0.5em\hbox{$\smash{\scriptscriptstyle\rightharpoonup}$}} {R} }_B}\left( {{t_1}} \right) - {{\overset{\lower0.5em\hbox{$\smash{\scriptscriptstyle\rightharpoonup}$}} {R} }_A}\left( {{t_1} - \Delta {t_1}} \right)} \right|+c \cdot \left[ {d{t_B}\left( {{t_1}} \right) - d{t_A}\left( {{t_1} - \Delta {t_1}} \right)} \right] \\ +c \cdot \left( {\delta _{B}^{{rec}}+\delta _{A}^{{send}}} \right)+{\Delta _{AB}}+{\varepsilon _{AB}} \\ {P_{BA}}\left( {{t_2}} \right)=\left| {{{\overset{\lower0.5em\hbox{$\smash{\scriptscriptstyle\rightharpoonup}$}} {R} }_A}\left( {{t_2}} \right) - {{\overset{\lower0.5em\hbox{$\smash{\scriptscriptstyle\rightharpoonup}$}} {R} }_B}\left( {{t_2} - \Delta {t_2}} \right)} \right|+c \cdot \left[ {d{t_A}\left( {{t_2}} \right) - d{t_B}\left( {{t_2} - \Delta {t_2}} \right)} \right] \\ +c \cdot \left( {\delta _{A}^{{rec}}+\delta _{B}^{{send}}} \right)+{\Delta _{BA}}+{\varepsilon _{BA}} \\ \end{gathered}$$

where the pseudorange observation ${P_{AB}}\left( {{t_1}} \right)$ is the Ka-band pseudorange measurement obtained when Satellite B receives the signal from Satellite A at time ${t_1}$, ${P_{BA}}\left( {{t_2}} \right)$ is the pseudorange observation generated when Satellite A receives the signal from Satellite B at time ${t_2}$, ${\vec {R}_A}\left( {{t_2}} \right)$ and ${\vec {R}_B}\left( {{t_1}} \right)$ represent the position vectors of Satellites A and B, respectively, $\Delta {t_1}$and $\Delta {t_2}$ represent the signal propagation times,* c* denotes the speed of light, $d{t_A}$ and $d{t_B}$ are the clock offsets of the respective satellites, $\delta _{{}}^{{send}}$and $\delta _{{}}^{{rec}}$ represent the signal transmission and reception delays due to the ISL equipment, ${\Delta _{AB}}$ and ${\Delta _{BA}}$ are the systematic error corrections, which include antenna phase center corrections, relativistic effects, multipath effects, and range measurement biases, ${\varepsilon _{AB}}$ and ${\varepsilon _{BA}}$ represent the observation noises. By combining the two-way observations, a CF combination observation is formed. Prior to the combination, the two-way ranging epochs must be normalized. The observations at times ${t_1}$ and ${t_2}$ are normalized to the reference epoch ${t_0}$:

2 $$\begin{gathered} {P_{AB}}\left( {{t_0}} \right)={P_{AB}}\left( {{t_1}} \right)+\Delta {P_{AB}} \\ =\left| {{{\vec {R}}_B}\left( {{t_0}} \right) - {{\vec {R}}_A}\left( {{t_0}} \right)} \right|+c \cdot \left[ {d{t_B}\left( {{t_0}} \right) - d{t_A}\left( {{t_0}} \right)} \right]+c \cdot \left( {\delta _{B}^{{rec}}+\delta _{A}^{{send}}} \right)+{{\tilde {\Delta }}_{AB}}+{\varepsilon _{AB}} \\ {P_{BA}}\left( {{t_0}} \right)={P_{BA}}\left( {{t_2}} \right)+\Delta {P_{BA}} \\ =\left| {{{\vec {R}}_A}\left( {{t_0}} \right) - {{\vec {R}}_B}\left( {{t_0}} \right)} \right|+c \cdot \left[ {d{t_A}\left( {{t_0}} \right) - d{t_B}\left( {{t_0}} \right)} \right]+c \cdot \left( {\delta _{A}^{{rec}}+\delta _{B}^{{send}}} \right)+{{\tilde {\Delta }}_{BA}}+{\varepsilon _{BA}} \\ \end{gathered}$$

where ${P_{AB}}\left( {{t_0}} \right)$ and ${P_{BA}}\left( {{t_0}} \right)$ represent the Ka-band virtual pseudorange observations corresponding to the normalized epoch ${t_0}$, ${\vec {R}_A}\left( {{t_0}} \right)$ and ${\vec {R}_B}\left( {{t_0}} \right)$ are the satellite position vectors at ${t_0}$, $d{t_A}\left( {{t_0}} \right)$ and $d{t_B}\left( {{t_0}} \right)$ are the satellite clock offsets at ${t_0}$,${\tilde {\Delta }_{AB}}$ and ${\tilde {\Delta }_{BA}}$ are the errors generated during the normalization process, $\Delta {P_{AB}}$ and $\Delta {P_{BA}}$ are the sums of the differences in inter-satellite distances and clock offsets between ${t_0}$ and ${t_1}$, ${t_0}$and ${t_2}$, respectively. Their expressions are given as follows:

3 $$\begin{gathered} \Delta {P_{AB}}=\left| {{{\vec {R}}_B}\left( {{t_0}} \right) - {{\vec {R}}_A}\left( {{t_0}} \right)} \right| - \left| {{{\vec {R}}_B}\left( {{t_1}} \right) - {{\vec {R}}_A}\left( {{t_1} - \Delta {t_1}} \right)} \right| \\ +c \cdot \left[ {d{t_B}\left( {{t_0}} \right) - d{t_A}\left( {{t_0}} \right)} \right] - c \cdot \left[ {d{t_B}\left( {{t_1}} \right) - d{t_A}\left( {{t_1} - \Delta {t_1}} \right)} \right] \\ \Delta {P_{BA}}=\left| {{{\vec {R}}_A}\left( {{t_0}} \right) - {{\vec {R}}_B}\left( {{t_0}} \right)} \right| - \left| {{{\vec {R}}_B}\left( {{t_2}} \right) - {{\vec {R}}_B}\left( {{t_2} - \Delta {t_2}} \right)} \right| \\ +c \cdot \left[ {d{t_A}\left( {{t_0}} \right) - d{t_B}\left( {{t_0}} \right)} \right] - c \cdot \left[ {d{t_A}\left( {{t_2}} \right) - d{t_B}\left( {{t_2} - \Delta {t_2}} \right)} \right] \\ \end{gathered}$$

The accuracy of the normalization corrections directly impacts the quality of the two-way combined observations, which primarily depends on the accuracy of the velocity and clock rates of the BeiDou satellites. In this study, the broadcast ephemeris of BeiDou satellites is utilized to compute their a priori positions, velocities, and clock offsets. The CF combination,${P_{CF}}\left( {{t_0}} \right)$, is obtained by summing ${P_{AB}}\left( {{t_0}} \right)$ and ${P_{BA}}\left( {{t_0}} \right)$. The expression for ${P_{CF}}\left( {{t_0}} \right)$ is given as follows:

4 $$\begin{gathered} {P_{CF}}\left( {{t_0}} \right)=\frac{{{P_{AB}}\left( {{t_0}} \right)+{P_{BA}}\left( {{t_0}} \right)}}{2} \\ =\left| {{{\vec {R}}_B}\left( {{t_0}} \right) - {{\vec {R}}_A}\left( {{t_0}} \right)} \right|+c \cdot \left( {{\delta _A}+{\delta _B}} \right)+\frac{{{{\tilde {\Delta }}_{AB}}+{{\tilde {\Delta }}_{BA}}}}{2}+{\varepsilon _{CF}} \\ \end{gathered}$$

where $\delta _{A}^{{}}=\frac{{\delta _{A}^{{send}}+\delta _{A}^{{rec}}}}{2}$ and $\delta _{B}^{{}}=\frac{{\delta _{B}^{{send}}+\delta _{B}^{{rec}}}}{2}$ represent half the sum of the transmission and reception hardware delays for the ISL equipment of Satellites A and B, respectively. During the computation of the satellite orbits, the orbital parameters, solar radiation pressure (SRP) model parameters, and ISL hardware delay parameters are estimated concurrently. This study focuses on the segmented estimation strategy for ISL hardware delays and its impact on orbit determination accuracy.

### Hardware delay variations and Estimation

The hardware delay in inter-satellite observations varies slowly and has been treated as a constant in existing research, with only one CF combination hardware delay estimated for each satellite over a 72-hour period. However, measurement noise differs among the links, and some links exhibit significant noise levels^19^, which can affect the estimation of hardware delays and orbital parameters. To address this, a segmented estimation approach is employed for the hardware delays of individual links to mitigate the impact of link noise or unmodeled errors on orbit determination, and to analyze the temporal variations of the hardware delays. The STD of the hardware delays for each link is calculated to quantify the time-varying characteristics of the hardware delays. This analysis aids in achieving a more comprehensive understanding of the stability and reliability of ISL signals, providing essential references for future applications. The expression for the link hardware delay is given as follows:

5 $$c \cdot \left( {{\delta _A}+{\delta _B}} \right)={P_{CF}}\left( {{t_0}} \right) - \left| {{{\vec {R}}_B}\left( {{t_0}} \right) - {{\vec {R}}_A}\left( {{t_0}} \right)} \right| - \frac{{{{\tilde {\Delta }}_{AB}}+{{\tilde {\Delta }}_{BA}}}}{2} - {\varepsilon _{CF}}$$

where $c \cdot \left( {{\delta _A}+{\delta _B}} \right)$ is hardware delay. To avoid the influence of orbital errors on the calculation of hardware delays, the experiment utilized precise ephemeris data released by the international GNSS Monitoring & Assessment System (iGMAS) Analysis Center of the Innovation Academy for Precision Measurement Science and Technology (APM), Chinese Academy of Sciences, to compute the accurate positions of BDS-3 satellites.

This study analyzed the variations in hardware delays for 135 links over a 45-day period from April 26 to June 9, 2020, and calculated the STD of the hardware delays for each link. After removing outliers exceeding three times the STD, over 95% of data remained for all links. Most links retained over 96% of data, indicating high-quality inter-satellite observation data. Some links like C19-C25 and C20-C21 showed slow delay variations and low STD values. However, links such as C28-C23, C28-C32, C30-C23, and C30-C33 exhibited noticeable delay jumps and higher STD values.

**Fig. 1:**
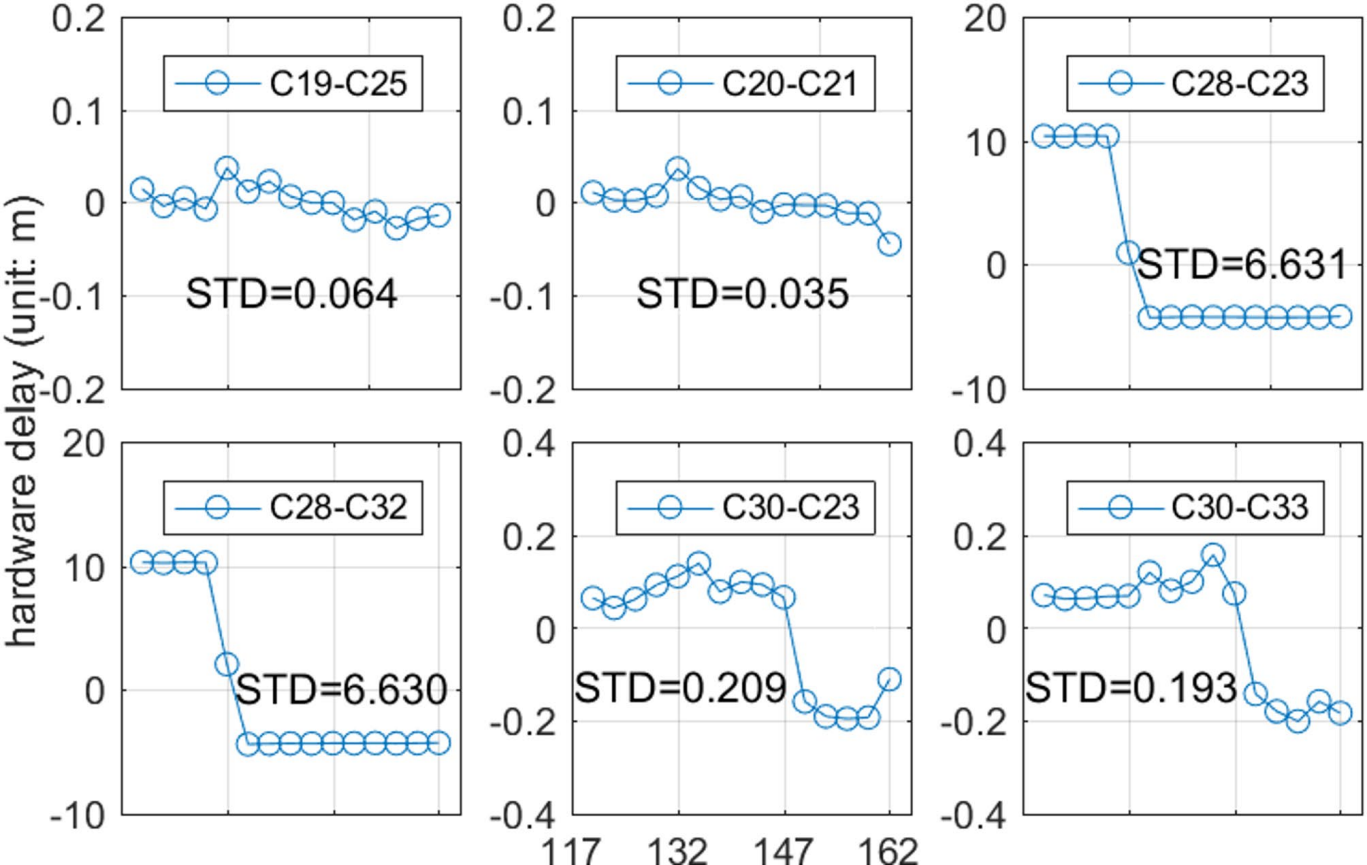
hardware delay variation of six links within 117 to 162 days in 2020. Each data point in the figure corresponds to the estimated hardware delay for a single ISL, calculated using a 72-hour arc length.

Figure 1 shows the hardware delay variations of six ISLs over 72-hour intervals. Assuming link hardware delays were constant during each interval, parameters were adjusted by subtracting their mean values. Results show that C19-C25 and C20-C21 delays varied slowly with STDs of 0.064 m and 0.036 m, indicating stability. Conversely, C28-C23, C28-C32, C30-C23, and C30-C33 showed significant variations. Their STDs were 6.631 m, 6.630 m, 0.209 m, and 0.193 m. C28-C32 and C28-C23 had larger jumps of about 15 m, while C30-C23 and C30-C33 had smaller jumps of 0.25 m.

**Fig. 2:**
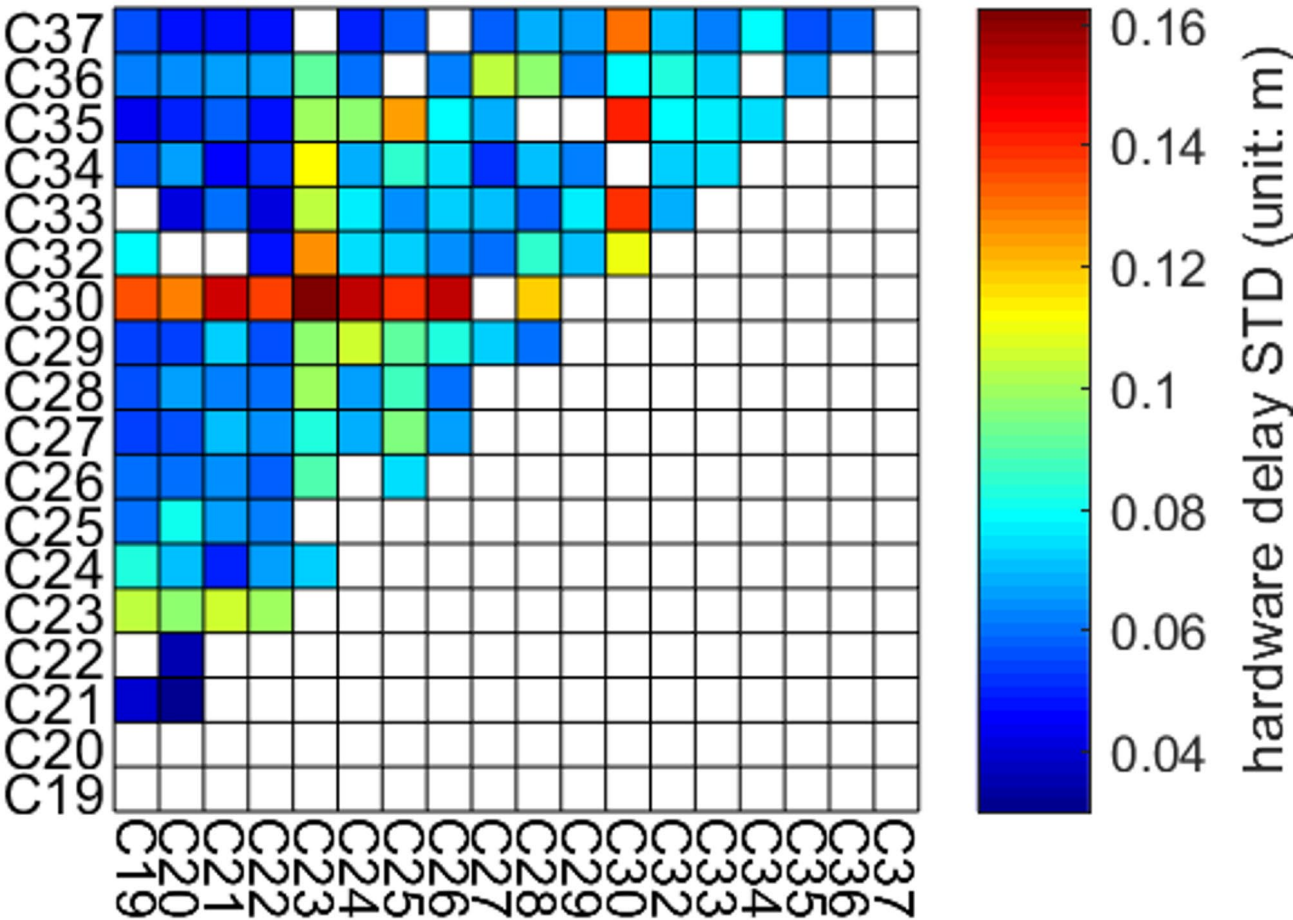
STD of hardware-bias variation within 117 to 162 days in 2020 (3-days arc), excluding hardware-delay jump of C28 and C30.

Figure 2 shows the STD values of hardware delay variations for all links, excluding jumps in C23, C28, and C30 links. Most links have STD values between 2.0 cm and 8.0 cm, showing no significant fluctuations. After removing hardware delay jumps, C28-related links exhibited good stability. However, C30-related links still had large STD values, mostly over 10.0 cm and up to about 16.0 cm.C23-related links also showed larger variations, with STD values around 10.0 cm. The C25-C35 link stands out with a relatively high STD of about 12 cm, yet other links involving C25 and C35 were of good quality, indicating varying quality among different ISLs.

**Fig. 3:**
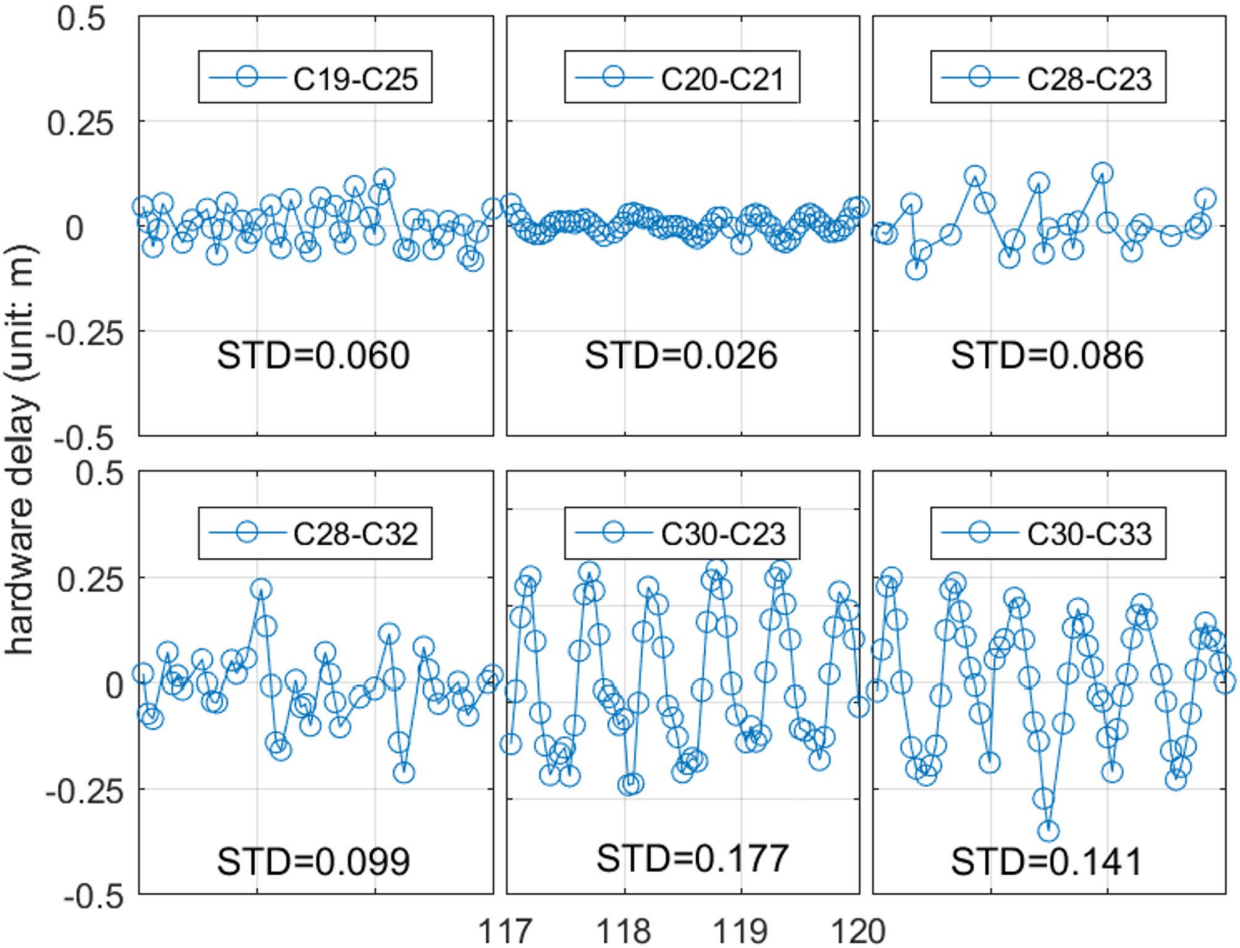
hardware delay variation of 6 links within 117 to 120 days in 2020. Each point in the figure represents the estimated hardware delay for a single ISL, calculated using a 1-hour arc length.

**Fig. 4:**
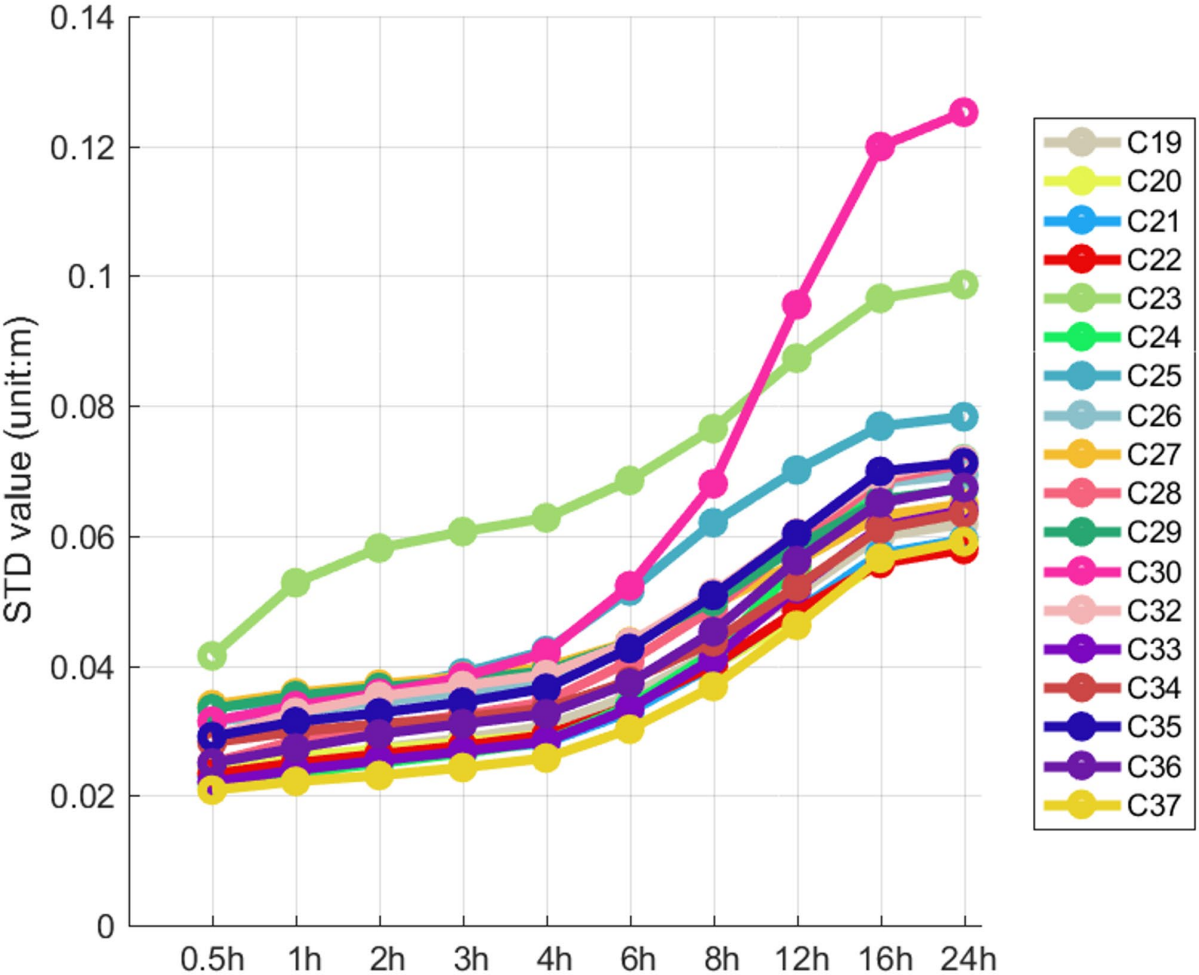
The average STD value of CF combination for different strategies.

Figure 3 shows the hardware delay variations for six ISLs over 72 h, with parameters estimated hourly. The C19-C25 and C20-C21 links show small STD values of 6.0 cm and 2.6 cm, while the C30-C23 and C30-C33 links have larger values of 17.7 cm and 14.1 cm. The other links also varied but without a clear pattern. The variations in hardware delays across different links are heterogeneous due to factors like equipment characteristics, satellite positions, and environmental conditions. To address the challenge of uniformly modeling these variations, a segmented estimation approach is proposed. Each link is divided into multiple segments with a hardware delay estimated for each. An optimal segmented strategy is determined by designing ten strategies with sampling intervals from 0.5 to 24 h. For each link, the STD of the hardware delay variation sequence is calculated for each arc segment. By analyzing how the STD changes with arc length, we discuss the characteristics of hardware delay variations under different strategies. Figure 4 presents the average STD for each set of links established by a single satellite under a given strategy. This aids in analyzing the impact of arc length selection on hardware delay estimation and provides a basis for optimizing link performance.

Figure 4 illustrates the variations in the STD values of the hardware delays for BDS-3 MEO satellite ISLs under various strategies. It is evident from the figure that there is a correlation between the STD values of the hardware delays and the arc segment lengths. As the arc segment length increases, the STD values of the hardware delays also tend to increase, with a consistent trend observed across different satellites. This phenomenon indicates a positive correlation between the STD values of the hardware delays and the arc segment lengths. In the schemes with arc lengths of 6 h, 8 h, 12 h, 16 h, and 24 h, the STD values of the hardware delays increase with the growth of the arc length. Conversely, in the schemes with arc lengths of 0.5 h, 1 h, 2 h, 3 h, and 4 h, the STD values of the hardware delays for most satellites change slowly.

In particular, Satellite C30 shows larger STD values than most other satellites in schemes with arc lengths beyond 4 h. Satellite C23 also differs from others, but less than C30.The analysis suggests most satellites have similar STD values under the same arc length scheme. As arc length increases, the STD values also rise. When the arc length is 24 h, the STD values of the links are all greater than 6 cm. In contrast, in schemes with arc lengths of 0.5–4 h, the STD values for most satellites stabilize within 2–4 cm, except for Satellite C23. Thus, in ISL-based POD, we can choose appropriate segmented strategies to estimate hardware delay parameters as needed.

To further analyze the link characteristics associated with satellites C23 and C30, a transformation was applied to the GF combination formula of the ISLs:

6 $$c \cdot \left[ {d{t_B}\left( {{t_0}} \right) - d{t_A}\left( {{t_0}} \right)} \right]=\frac{{{P_{AB}}\left( {{t_0}} \right) - {P_{BA}}\left( {{t_0}} \right)}}{2} - c \cdot \left( {\delta _{A}^{\prime } - \delta _{B}^{\prime }} \right) - \frac{{{{\tilde {\Delta }}_{AB}} - {{\tilde {\Delta }}_{BA}}}}{2} - {\varepsilon _{GF}}$$

where $c \cdot \left[ {d{t_B}\left( {{t_0}} \right) - d{t_A}\left( {{t_0}} \right)} \right]$ represents the difference in clock offsets between satellite A and satellite B at time ${t_0}$, also referred to as the relative clock offset. The variation in satellite clock offsets from the broadcast ephemeris exhibits a linear correlation with time over short durations. By performing a linear fitting on the relative clock offsets derived from the GF combination, the residuals obtained after removing the trend term can effectively reflect the measurement noise characteristics of the link.

**Table 1:** Information on manufacturers of BeiDou-3 ISL equipment.

Manufacturers of ISL Equipment	PRN
CASC1	C19-C22, C24, C33, C37-C42, C46-C61
CASC2	C23, C32, C36, C45
SECM1	C27, C29, C30, C34, C35, C43, C44
SECM2	C25, C26
SECM3	C28

To facilitate the analysis of the impact of link equipment on its measurement characteristics, the satellite PRN numbers are sorted based on the manufacturer information of the link equipment listed in Table 1. Additionally, the fitting residual plot of the GF combination is generated for further examination.

**Fig. 5:**
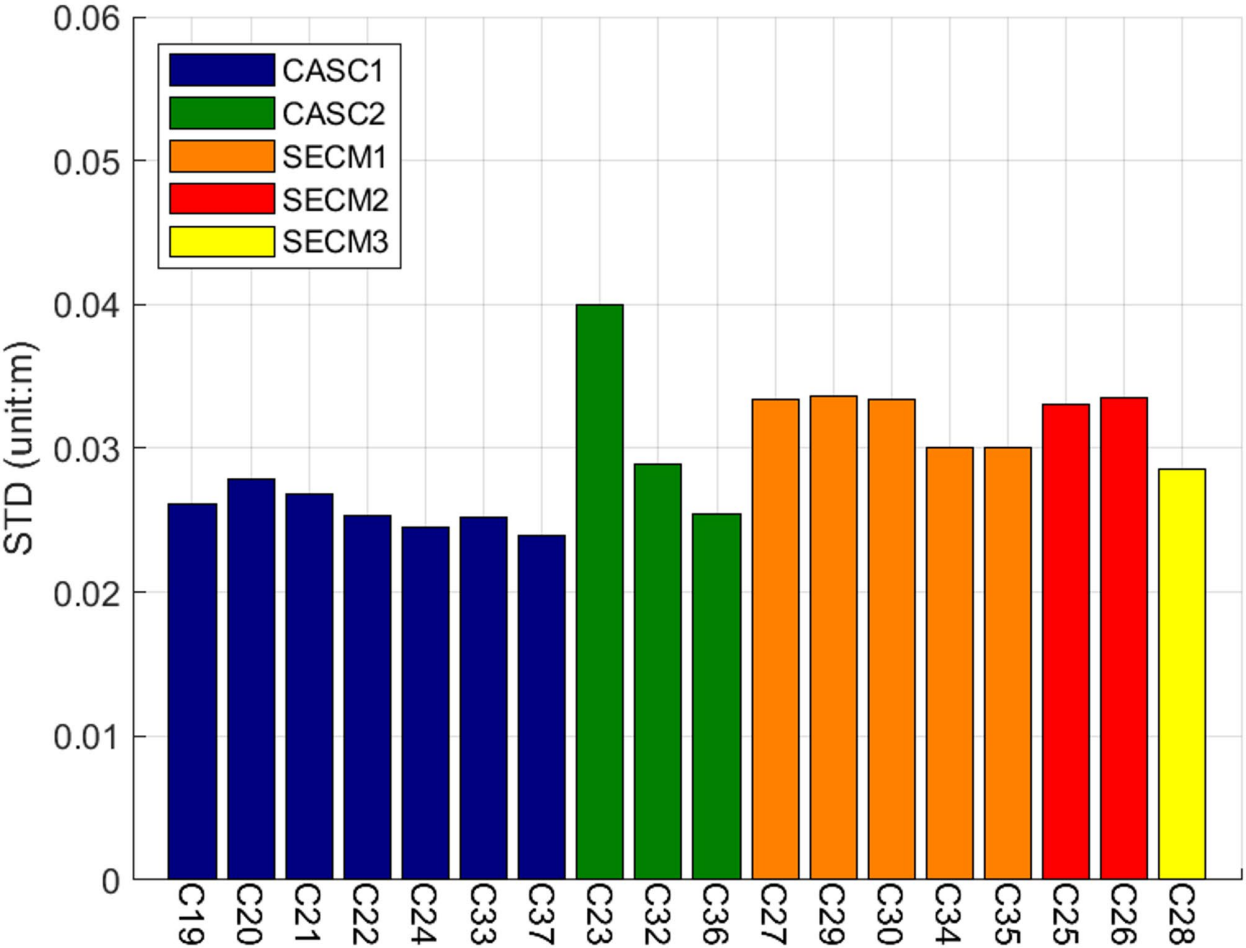
STD Values of Linear Fitting Residuals for GF Combinations Related to Satellites.

**Fig. 6:**
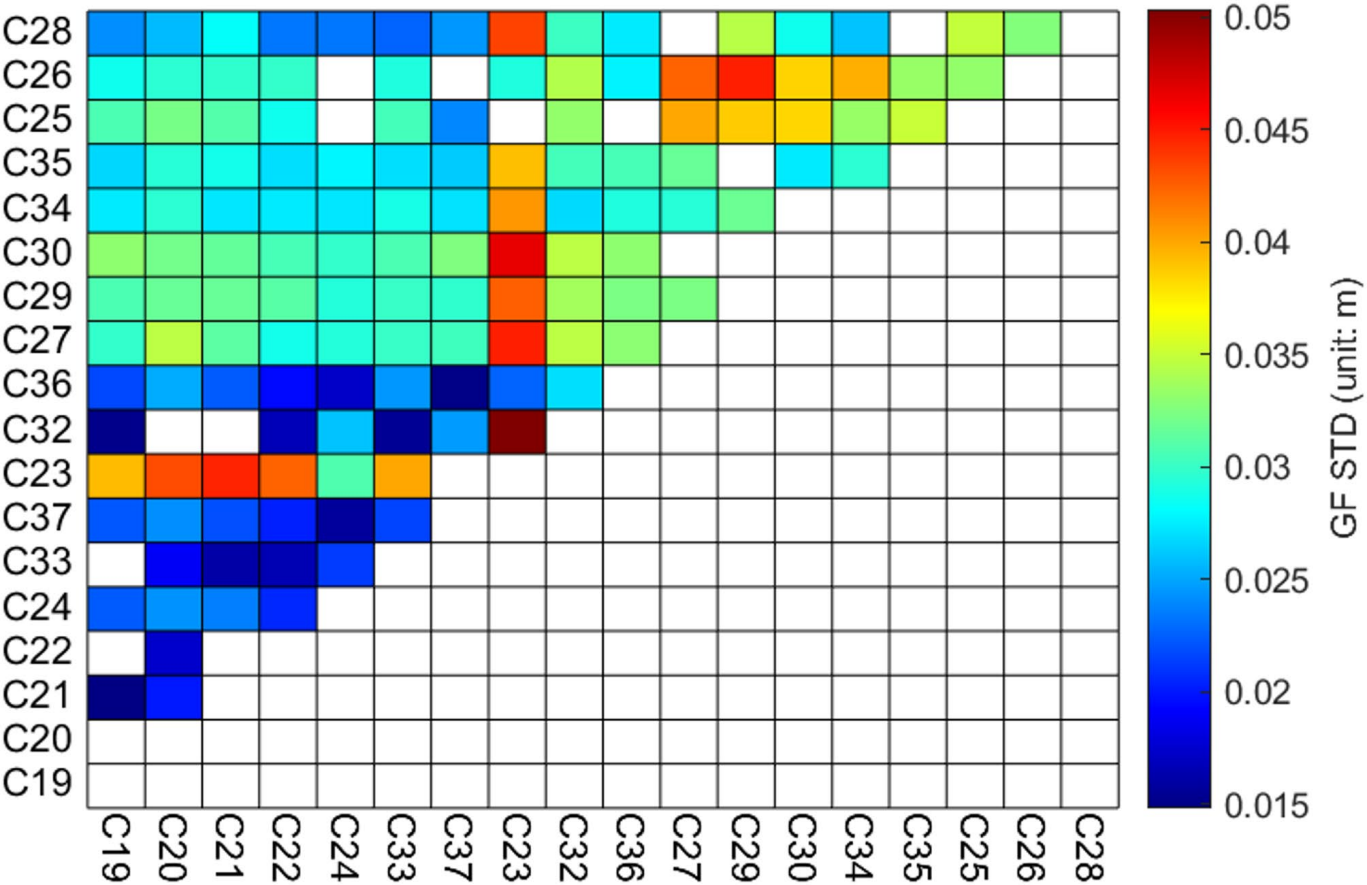
STD Values of Linear Fitting Residuals for GF Combinations in ISL.

Figure 5 displays the STD values of the GF combination linear fitting residuals for inter-satellite link equipment manufactured by five providers: CASC1, CASC2, SECM1, SECM2, and SECM3. A correlation is observed between the link equipment and the fitting residuals of the GF combination. The noise levels vary significantly among different equipment. Among all satellites, satellite C23 exhibits the highest noise level, followed by C30. When satellite C23, which has the highest noise level, is excluded from the analysis, the link equipment from CASC1, CASC2, and SECM3 demonstrates lower noise levels compared to that from SECM1 and SECM2. To further investigate the influence of different equipment on the noise characteristics of the GF combination, all links are grouped according to their respective manufacturers, and the corresponding STD value distribution of the GF combination is plotted, as shown in Fig. 6.

Figure 6 illustrates the distribution of the STD values of the GF combination linear fitting residuals for the links, sorted by equipment manufacturer. A notable block-like pattern is evident in Fig. 6, indicating that significant differences exist in the link noise levels among different equipment types. This further confirms the correlation between link noise and the associated equipment. Among the devices produced by the five manufacturers, links established using equipment from CASC1 and CASC2 exhibit relatively lower noise levels. In contrast, links constructed with SECM1 and SECM2 equipment demonstrate significantly higher noise levels compared to other links, suggesting that the stability of SECM1 and SECM2 equipment is slightly lower than that of the others. Additionally, most links associated with satellite C23 exhibit STD values higher than those of other links.

**Table 2:** STD values of linear fitting residuals for GF combinations related to link equipment (unit: cm).

Equipment Type	CASC1	CASC2	SECM1	SECM2
CASC1	2.10			
CASC2	2.09	2.57		
SECM1	2.78	2.85	3.14	
SECM2	2.97	3.00	3.67	3.65
SECM3	2.46	2.81	2.73	3.39

Table 2 presents the STD values of the GF combination linear fitting residuals for different equipment types. Links associated with CASC1-CASC1 and CASC2-CASC2 exhibit lower noise levels, with STD values of 2.10 cm and 2.09 cm, respectively. In contrast, links related to SECM1-SECM2, SECM2-SECM2, and SECM2-SECM3 demonstrate higher noise levels, with STD values of 3.67 cm, 3.65 cm, and 3.39 cm, respectively. This further confirms that links involving SECM1 and SECM2 are associated with significantly higher noise levels.

Based on the analysis of the GF combination linear fitting residuals, it can be concluded that the noise in the inter-satellite link GF combination exhibits a strong correlation with the link equipment. The interconnection of link equipment manufactured by different manufacturers results in variations in the noise levels of the links. Notably, the noise levels of links associated with satellite C23 are significantly higher than those of other links. To analyze the impact of equipment differences on the CF combination, the satellites are grouped according to their link equipment, and a statistical plot of the hardware delay variations in the CF combination is generated.

**Fig. 7:**
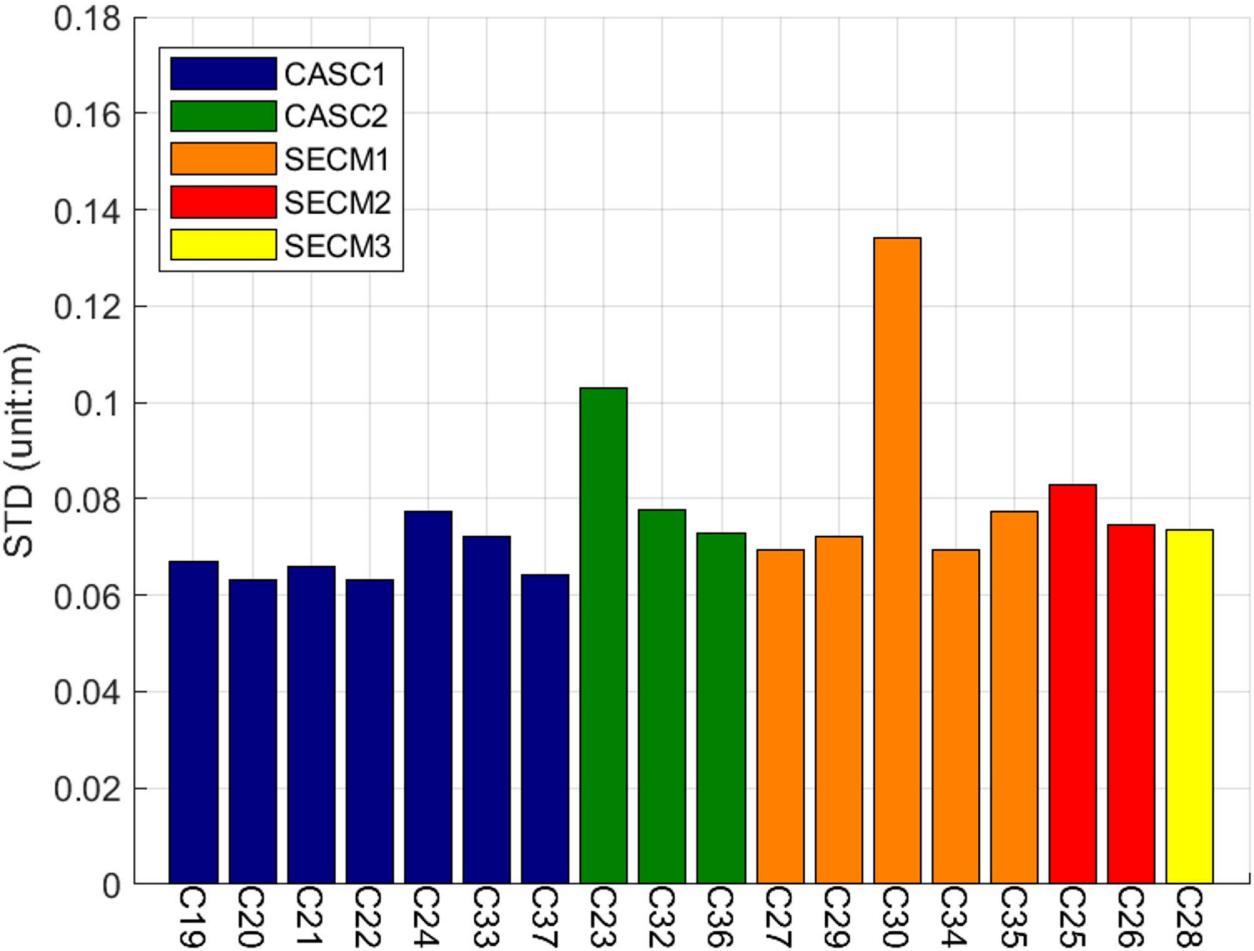
STD Values of Hardware Delay for CF Combinations Related to Satellites.

**Fig. 8:**
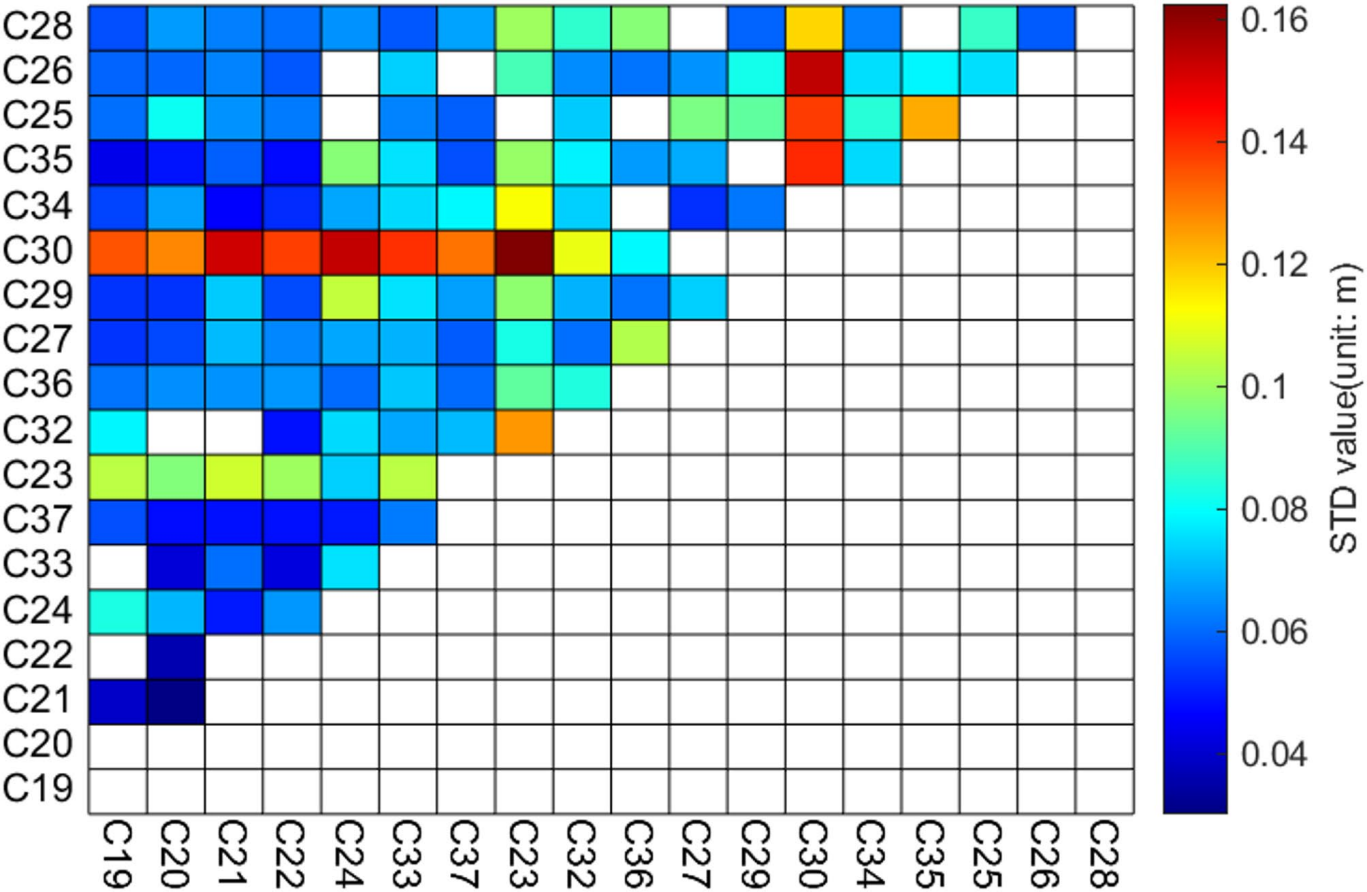
STD Values of Hardware Delay for CF Combination in ISL (Satellite PRNs Sorted by Link Equipment Manufacturers).

Figure 7 illustrates STD values of the hardware delays in the CF combination for each satellite. Among the 18 MEO satellites, the CF combination STD values of satellites C23 and C30 are notably higher than those of the other satellites, with their respective STD values being 10.30 cm and 13.41 cm. Excluding satellites C23 and C30, no significant differences were observed in the STD values of the remaining satellites. The smallest STD value was associated with satellite C22, measuring 6.31 cm, while the largest STD value was recorded for satellite C25 at 8.29 cm. To further investigate the influence of equipment differences on the CF combination of individual links, the links were grouped according to their corresponding equipment types, and the distribution of hardware delay variations in the CF combination was replotted accordingly (see Fig. 8).

Figure 8 presents the distribution of STD values of hardware delays in the CF combination, grouped by equipment type. It is evident that, excluding satellites C23 and C30, there are no significant differences in the CF combination hardware delay STD values among different equipment types, indicating the absence of a pronounced block-like pattern. However, both the GF and CF combination STD values for satellite C23 are higher than those for other satellites, suggesting that the measurement noise or variations in hardware delays for the link equipment on C23 are relatively large. Although the GF combination STD value for satellite C30 does not significantly differ from those equipped with SECM1 and SECM2 devices, its CF combination STD value is notably higher. There are three potential reasons for this: first, the orbital errors of satellite C30 may be larger than those of other satellites, affecting the estimation of hardware delays in the CF combination and thus leading to a higher STD value; second, the hardware delays in the CF combination for C30 might exhibit greater variability; third, there could be unmodeled errors in the link equipment of C30. To address the first possibility, we evaluated the precise orbit provided by the APM analysis center using overlapping orbit comparisons and SLR, and found no significant difference in the orbital accuracy between C30 and other MEO satellites. This eliminates the first hypothesis. Due to space limitations, the overlap orbit and SLR validation results for C30 are not shown here. For the second and third hypotheses, given the current lack of internal inspection methods for satellite link equipment, it is not possible to directly determine which factor is the direct cause of the increased variability in the CF combination hardware delay estimates for satellite C30.

## ISL-based POD experiments and analysis

This section provides an overview of ISL establishment for BDS-3 and introduces POD method based on segmented estimation strategy for ISL hardware delays. Through systematic evaluations of orbital accuracy, this study aims to validate the effectiveness of the segmented estimation approach and its impact on enhancing the accuracy of POD for BDS-3 satellites.

### ISL experimental data

**Fig. 9:**
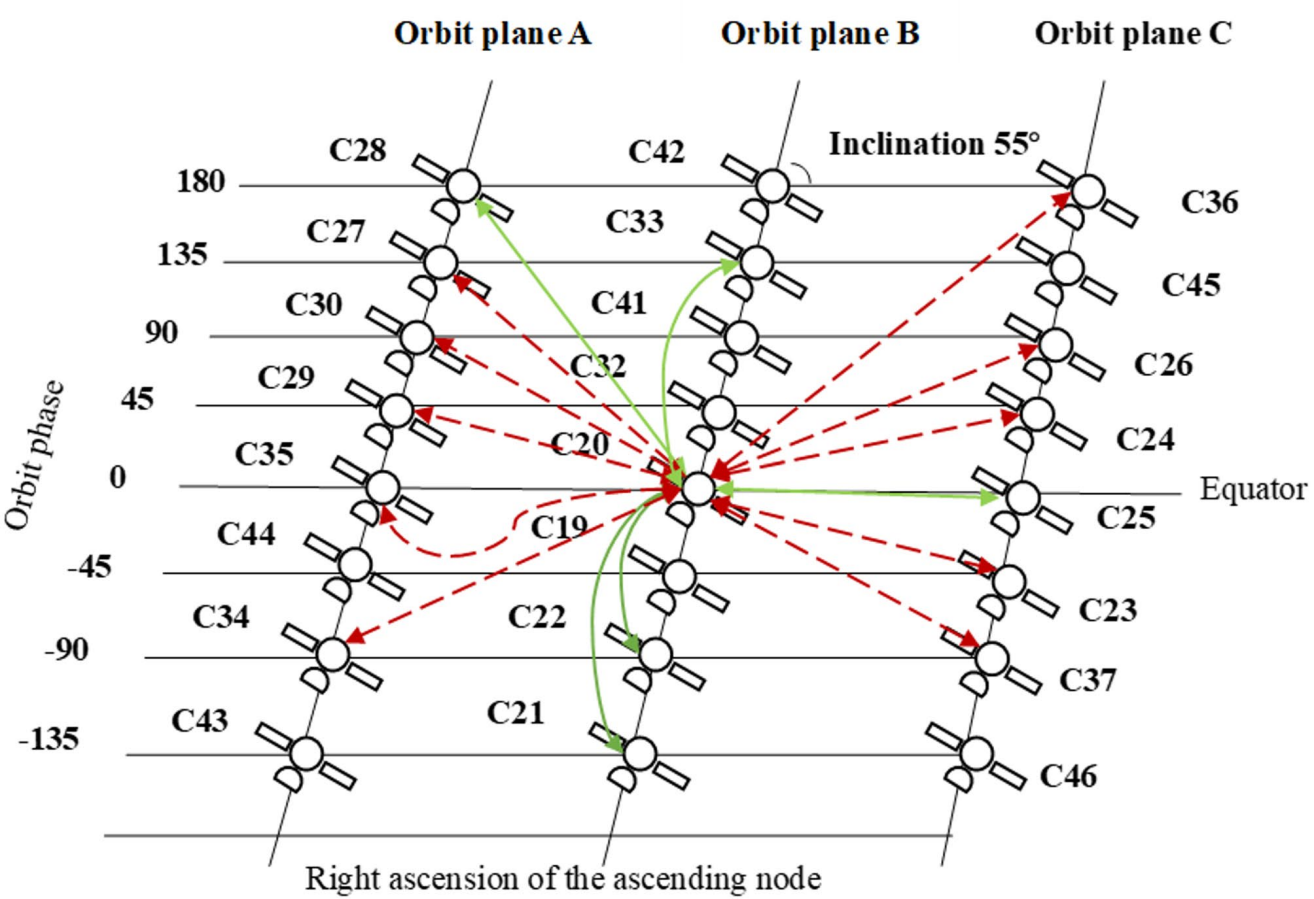
The diagram illustrates the ISLs establishment between MEO satellite C20 and other MEO satellites, where solid green lines represent continuous links and dashed red lines indicate intermittent links.

This study selected continuous ISL measurement data from BDS-3 MEO satellites over a 45-day period, from April 26 to June 9, 2020. The BDS-3 MEO satellites are primarily distributed across three orbital planes. Specifically, satellites C19, C20, C21, C22, C32, and C33 are in the second orbital plane; satellites C27, C28, C29, C30, C34, and C35 are in the first orbital plane; satellites C23, C24, C25, C26, C36, and C37 are in the third orbital plane. The distribution of BDS-3 MEO satellites allows the ISLs to be categorized into two types: Co-plane links and cross-plane links. Co-plane links refer to those established between satellites within the same orbital plane, whereas cross-plane links connect satellites residing in different orbital planes. Figure 9 illustrates the ISLs establishment between MEO satellite C20 and other MEO satellites. Within the selected timeframe, 135 links were established among 18 MEO satellites.

### Segmented estimation strategy for ISL hardware delays

In previously published research, the hardware delays of ISLs are considered constant over short periods, with a single hardware delay estimated for each satellite over a 72-hour interval. However, the hardware delays of a few links can vary significantly over such periods. The analysis of ISL hardware delays demonstrates that segmenting the estimation of ISL hardware delays can effectively reduce the STD of the CF combination. Therefore, in this section, different segmented strategies for estimating hardware delays are implemented and evaluated for their impact on the accuracy of ISL orbit determination.

Traditional methods typically estimate a constant hardware delay for each satellite over a 72-hour interval. However, considering the short-term fluctuations in hardware delay between satellite pairs in each link, this paper estimates a constant hardware delay for each link in the experiment, with different intervals of 1 h, 2 h, 3 h, 4 h, 6 h, 8 h, 12 h, 16 h, 24 h, and 72 h. For simplicity, the traditional method is abbreviated as “Satellite-specific-72 h” or “72 h-A”, while the method proposed in this paper is abbreviated as “Link-specific-*h” or “*h-B”, where * represents the different intervals.

The BDS-3 ISLs operate under a CSTDD system^5^. The term “Concurrent” refers to the ability of multiple satellites to establish connections simultaneously. The term “Spatial” indicates that satellites can switch their connection targets, enabling them to observe multiple other satellites. The term “Time Division” signifies that satellites perform observations at different times. Under the CSTDD system, a single satellite can observe multiple other satellites, but it can only observe one satellite at any given moment. This implies that among the multiple links established by a single satellite, there are no simultaneous observations. Additionally, raw ISL observations contain both satellite clock biases and hardware delays, which are linearly correlated and thus cannot be directly resolved. Consequently, we use prior satellite positions, prior clock biases, and Eq. (4) to construct a CF combination, which is then used for orbit determination. During the construction of the CF combination, it is necessary to convert the two-way observations at times ${t_1}$ and ${t_2}$ to time ${t_0}$, where ${t_0}$ is an integer second between ${t_1}$ and ${t_2}$^19^. In the data processing for this study, prior satellite positions and clock biases are provided by broadcast ephemerides.

**Table 3:** Options for ISL POD.

Type	Name	description
Observation model	ISL	Ka band ISL observations
dynamic model	N-body	Positions of Sun, moon, and other planets provided by JPL DE421^26,27^
	Solid earth tides	TIDE2000^28^
	Ocean tides:	OT_FES2014b^28^
	Earth’s gravitational model:	EGM2008_SMALL^28^
	Sub-daily pole:	IERS2010XY^28^
	Nutation	IAU2000R06^28^
	Earth orientation	CODE products
	Relativistic effects	IERS Conventions 2010^28^
estimated parameters	GNSS orbit parameters	Initial value provided by Broadcast ephemeris
	Solar pressure parameters	parameters of the five-parameter ECOM model ^29^
	Ka hardware delay parameters	hardware delay biases per satellite or per link
estimation method	Least Squares Method	Parameters estimated simultaneously include those for satellite orbits, solar radiation pressure, and hardware delays.
other models	Ka band PCO	manufacturer values for Ka antennas
	Attitude model	Yaw steering model for BDS-3 MEO satellites ^30^

In this study, the arc length for POD is set to 72 h. The process of orbit determination includes ISL data preprocessing, orbital integration, and orbit solution computation. Broadcast ephemerides are used to generate the equations of motion and the variational equations for the satellites. Table 3 provides details of options employed for orbit determination. The orbital mechanics models used in the data processing include the gravitational influences of the Sun, Moon, Jupiter, Venus, and Mars^26^, relativistic effects, polar tides, precession, and nutation^28^. In the process of orbit determination, broadcast ephemerides were initially employed to compute the preliminary orbital trajectory, while ISL observations were utilized to estimate the six orbital parameters, ECOM 5 solar radiation pressure model parameters, and ISL hardware delay parameters. Consequently, ground-based tracking stations were not directly employed in this methodology. In reality, broadcast ephemerides are generated based on observational data from ground tracking stations. The integration of broadcast ephemerides and ISL observations can be considered as an indirect utilization of ground station observational data.

### Orbit result analysis

We have designed various hardware delay estimation strategies (Table 3). This section primarily evaluates the accuracy of orbit determination under different hardware delay estimation strategies. The rest of this section discusses the ISL residuals and the reference orbit comparison results.

Residuals from ISL observations reflect the internal consistency of orbital accuracy. Using the various hardware delay estimation strategies detailed in Table 3, we processed the ISL data. By analyzing the distribution and RMS of the ISL POD residuals for the links, we can discern the differences among the different hardware delay estimation strategies.

**Table 4:** RMS values of ISL POD residuals for strategies ranging from 1 h-B to 72 h-B (Units: m).

Type	1 h	2 h	3 h	4 h	6 h	8 h	12 h	16 h	24 h	72 h
All	0.030	0.032	0.033	0.035	0.039	0.044	0.050	0.055	0.057	0.063
Exclude C23/C30	0.028	0.029	0.031	0.032	0.036	0.040	0.045	0.050	0.051	0.057

**Fig. 10:**
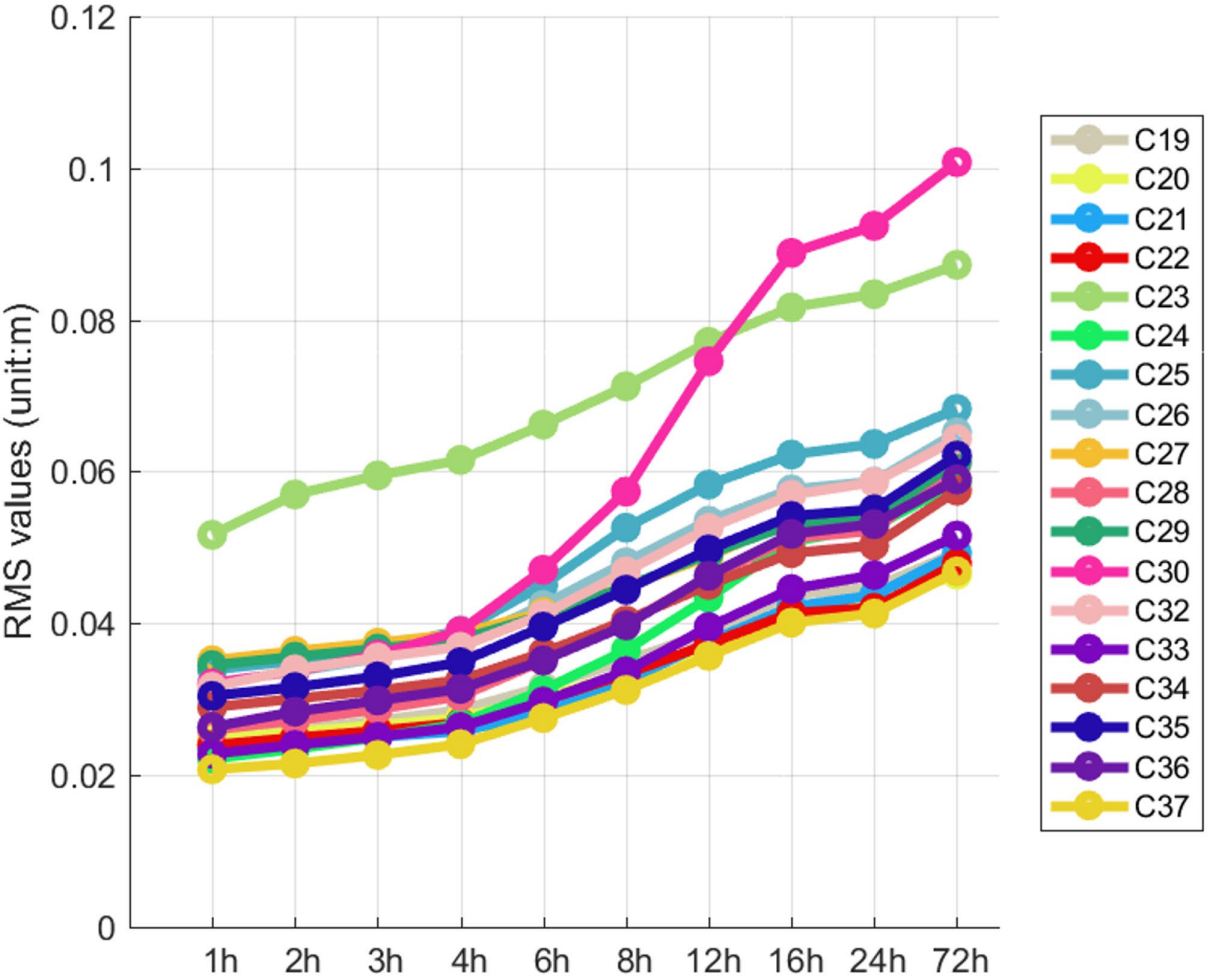
RMS of ISL POD residuals for 1 h-B to72h-B strategies of BDS-3 MEO satellites.

Table 4 presents the RMS values of the ISL POD residuals for strategies ranging from 1 h-B to 72 h-B. The data indicate a significant correlation between ISL residuals and the arc length of segmented hardware delay estimates: as the arc length increases, the RMS values of ISL residuals also increase accordingly. Furthermore, after excluding the data from satellites C23 and C30, a reduction in the ISL residual RMS values was observed. This suggests that segmenting the estimation of hardware delays for individual links better aligns with the characteristics of ISL observations. To provide a more intuitive illustration of the ISL POD residuals for all satellites, we have plotted the relationship between the RMS values of residuals and the arc length of segmented hardware delays for each satellite in a line graph (see Fig. 10).

Figure 10 shows the variations in the RMS values of ISL residuals for BDS-3 MEO satellites. Each point in the figure represents the RMS of the ISL POD residuals for all relevant links associated with a satellite. For instance, the point on the pink line at the 6 h mark on the x-axis indicates the RMS of the ISL POD residuals for all links related to satellite C30 under the 6 h-B strategy. The RMS values of ISL residuals correlate with the arc length of hardware delays, increasing as the arc length increases. This trend is consistent across most satellites, except for C23 and C30. For C23, the RMS values range from 5 cm to 9 cm, showing a linear trend and higher values compared to other satellites at shorter arc lengths (1–8 h). This suggests higher measurement noise in links involving C23. Satellite C30 exhibits the largest RMS fluctuations, varying between 3 cm and 10 cm. Under 1–4 h strategies, C30’s RMS values are stable and similar to other satellites. However, at 6–16 h strategies, the RMS values increase rapidly, indicating that C30’s hardware delays change slowly within a 4-hour period. When the sampling interval is less than 4 h, most satellites have RMS values within 2–4 cm, with C37 showing the smallest values at around 2 cm.

**Fig. 11:**
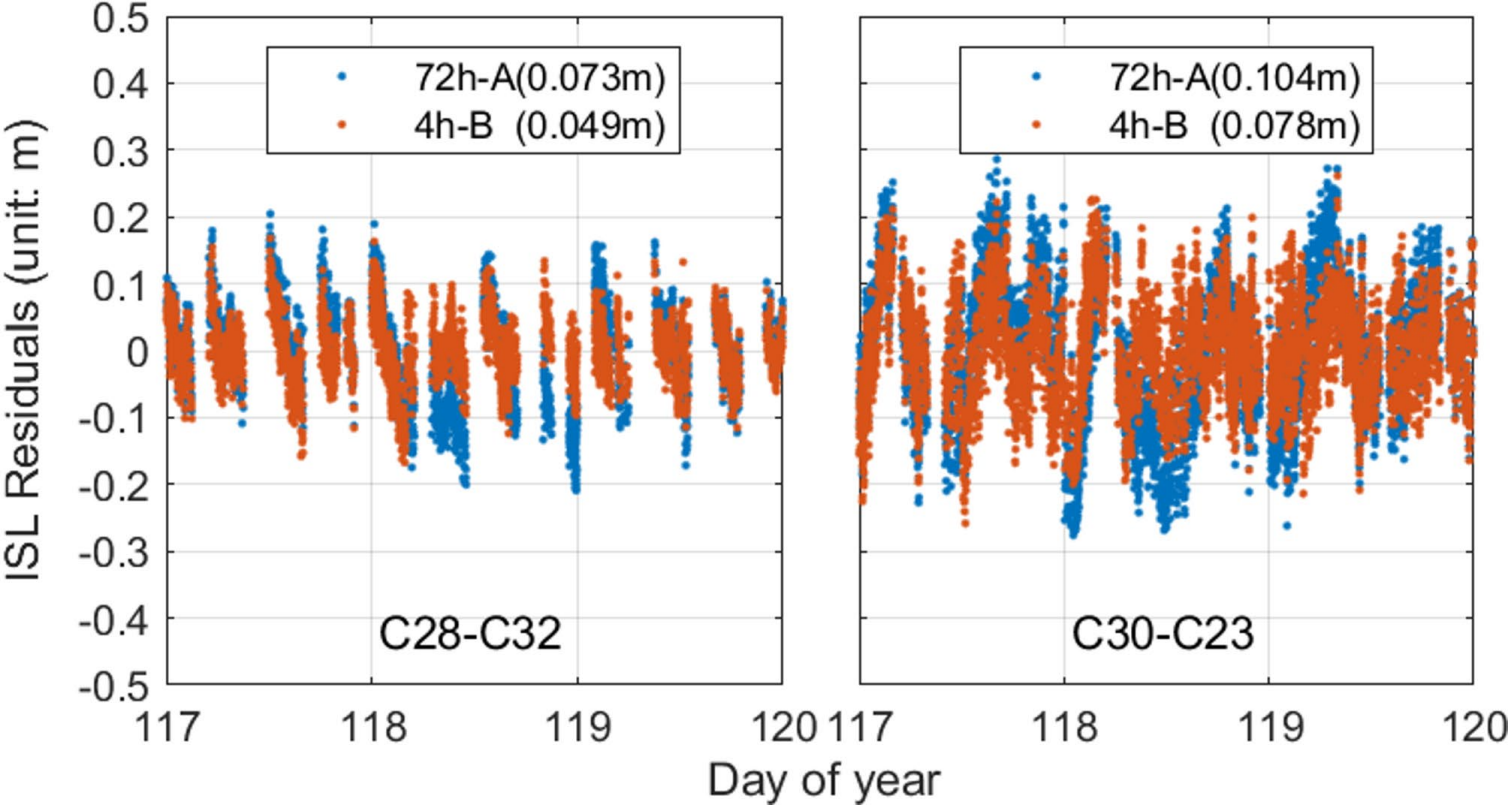
ISL POD residuals for traditional strategy(72 h-A) and our strategy(4 h-B); the parentheses in the legend show the RMS value of the residuals.

Figure 11 illustrates the ISL residuals for two links under the 72 h-A and 4 h-B strategies, respectively. Under the 72 h-A strategy, the RMS values of the residuals for the two links vary significantly, with residual values distributed between − 30.0 cm and 30.0 cm. The RMS values for C28-C32 and C30-C23 are close to 10 cm, with C30-C23 having the highest RMS value of 10.4 cm, indicating poorer observational data quality. Under the 4 h-B strategy, the RMS values for the two links, C28-C32 and C30-C23, show a substantial decrease, by 32.88% and 25.00%, respectively. Nonetheless, the RMS value for C30-C23 remains relatively high at 7.8 cm. This is due to the generally higher measurement noise associated with the links involving satellites C23 and C30. Overall, despite the varying degrees of improvement, the two links consistently indicate that a segmented estimation strategy is more suitable for links with significant hardware delay variations.

Comparing satellite orbits derived from different types of observational data is a method used to assess external orbit accuracy. IGS stations are capable of tracking GNSS satellites and collecting the carrier phase and pseudorange signals. L-band phase and pseudorange measurements achieve millimeter and meter-level precision, respectively, making them suitable for the POD of BDS-3 satellites. Analysis centers such as CNES/CLS, CODE, Wuhan University (WHU), German Research Centre for Geosciences (GFZ), and APM use observational data from IGS stations to generate precise orbit products for GNSS satellites. Currently, the precise orbit products provided by the IGS Analysis Center for BDS-3 satellites have a radial accuracy better than 5 cm^31,32^. However, due to limitations in ground visibility, L-band orbit determination requires dozens or even hundreds of globally distributed ground observation stations. In this study, we use the precise ephemeris released by the iGMAS Analysis Center as a reference for comparing with the ISL POD results.

**Fig. 12:**
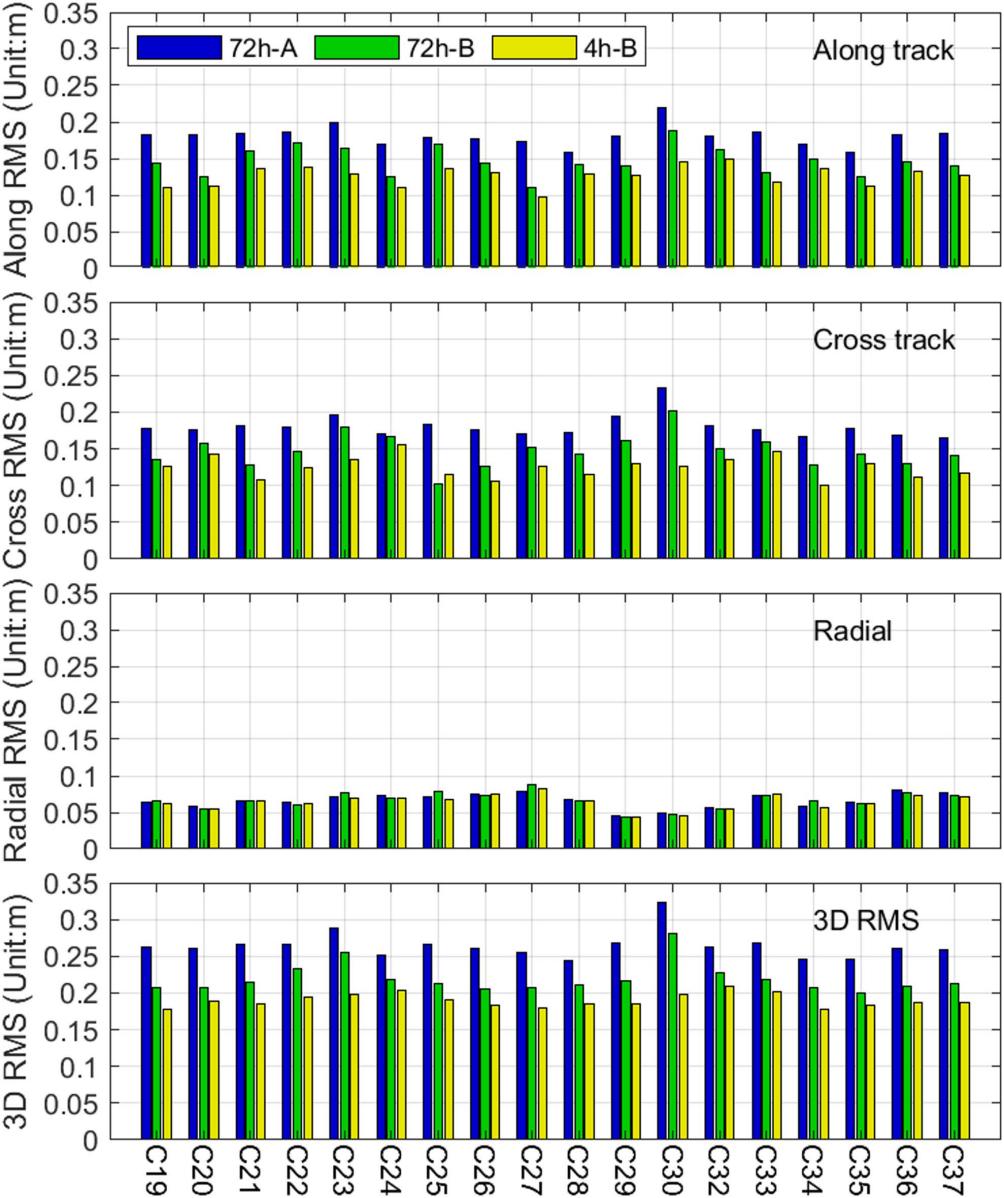
Along track, Cross track, Radial, and 3D RMS of differences between ISL and L-band orbits.

Figure 12 presents the RMS differences between ISL and L-band orbits under three strategies across different directions. The strategies rank in terms of 3D RMS as 72 h-A > 72 h-B > 4 h-B, indicating that segmented hardware delay estimation improves orbit determination accuracy. Under 72 h-A, satellites C23 and C30 exhibit higher 3D RMS values (28.9 cm and 32.3 cm, respectively) due to noisier links; however, these values decrease significantly under 4 h-B (19.88 cm and 19.75 cm, respectively), representing reductions of 31.2% and 38.9%. Radial direction RMS differences are minimal among the strategies, while along-track and cross-track directions show significant variations and marked decreases in RMS values as the segmented arc length shortens.

SLR validation is a method used to assess the external orbit accuracy of satellites. We calculate SLR residuals by subtracting the geometric distance from the satellite to the SLR ground station from the SLR observations, using the coordinates of the SLR stations provided by the International Laser Ranging Service. In this study, we apply the MENDES-PAVLIS model to correct for tropospheric delays^33^, and we use SLR Normal Point (NP) data from May 1, 2020, to May 31, 2020, to validate the POD results (https://cddis.nasa.gov/archive/slr/data/fr_crd/quarantine/).

**Fig. 13:**
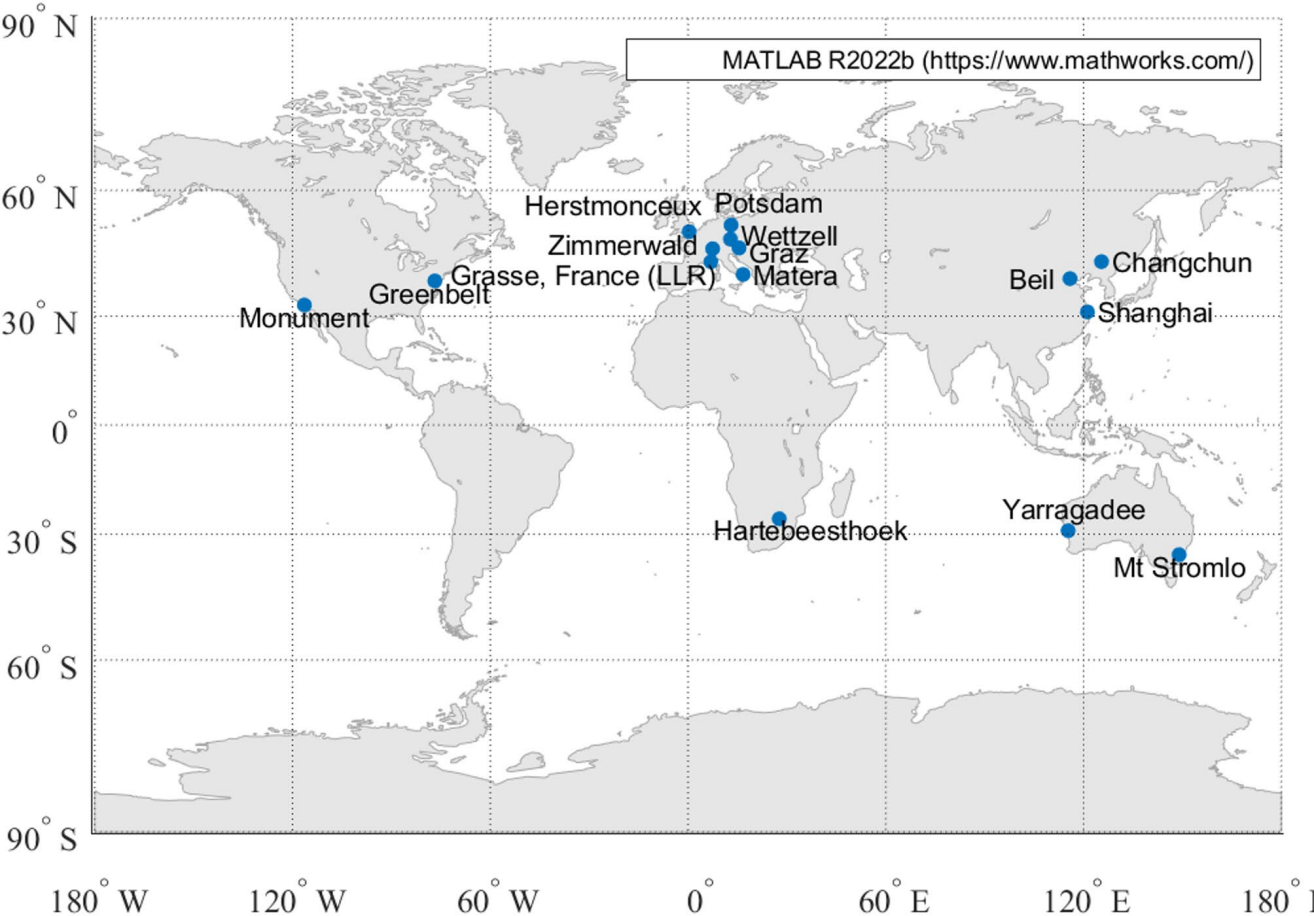
SLR stations distribution.

Figure 13 shows the distribution of SLR stations observing BDS-3 MEO satellites, involving a total of 15 stations. After excluding data with residuals exceeding 30 cm, significant differences in data volume among stations were observed. Yarragadee contributed the most (335 NP points, with 99.70% having residuals within 30 cm), whereas Hartebeesthoek contributed the least (only 4 data points). Overall, 1,286 NP points were recorded, 99.77% of which had residuals under 30 cm, demonstrating high data quality.

**Table 5:** SLR residuals information (units: cm).

PRN	STD		RMS		NP numbers
	4 h-B	72 h-A	4 h-B	72 h-A	
C20	2.89	3.37	4.59	5.30	327
C21	2.60	2.86	5.69	6.22	417
C29	4.11	4.30	4.19	4.38	280
C30	3.81	4.26	4.21	4.71	259

Table 5 shows the SLR residuals statistics for satellites C20, C21, C29, and C30 under the 4 h-B and 72 h-A strategies. All four satellites have over 250 NP points, ranging from 259 (C30) to 417 (C21). Under 4 h-B, STD values are under 5 cm, with C21 at 2.60 cm and C29 at 4.11 cm, indicating good orbit determination accuracy. Compared to 72 h-A, 4 h-B yields lower STD values for all four satellites, with C20 showing the largest decrease (14.31%) and C29 the smallest (4.40%). SLR validation confirms that segmented hardware delay estimation per link effectively improves orbit determination accuracy.

## Summary and conclusion

ISLs significantly enhance the orbit determination accuracy of BDS-3 satellites. Prior studies assumed constant hardware delays for CF-combined observations over 72 h, estimating a single parameter per satellite. However, analysis reveals substantial variations in certain links, notably those involving satellites C23 and C30, indicating that hardware delay changes impact accuracy. We propose a segmented estimation strategy for individual link hardware delays, evaluated using POD residual analysis, L-band orbit comparisons, and SLR validation. In the internal accuracy assessment, 4-hour arc segmentation of ISL hardware delays reduces POD residual RMS values to less than 4 cm for most ISLs, a 1–3 cm improvement over traditional methods. In external accuracy assessments, L-band and ISL orbit differences are at the decimeter level, with a significant decrease in 3D RMS values under the segmented strategy. SLR residual STD and RMS values are also lower, with an overall difference of approximately 0.5 cm. For satellites C23 and C30, POD residuals decrease from 8.7 cm to 10.1 cm to 6.2 cm and 3.9 cm, respectively, and 3D RMS differences with L-band orbits decrease from 28.9 cm to 32.3 cm to 19.9 cm and 19.8 cm, respectively. These satellites show more pronounced improvements in orbit accuracy compared to other BDS-3 MEO satellites.

In conclusion, the segmented estimation strategy aligns closely with the actual characteristics of ISL hardware delays, effectively mitigating their impact on BDS-3 orbit determination. This strategy is particularly beneficial for satellites C23 and C30, significantly enhancing their orbit determination accuracy.

## Data Availability

Data and materials for this article are available upon request from the corresponding author, Yunbin Yuan.
